# The implementation of a zero-suicide framework in a child and youth mental health service in Australia: processes and learnings

**DOI:** 10.3389/fpsyt.2024.1370256

**Published:** 2024-05-16

**Authors:** Grace Branjerdporn, Laura K. McCosker, Derek Jackson, Sarah McDowell, Philip Williams, Sandeep Chand, Hitesh Joshi, Anthony R. Pisani, Chris Stapelberg, Matthew Welch, Kathryn Turner, Sabine Woerwag-Mehta

**Affiliations:** ^1^ Mental Health and Specialist Services, Gold Coast Hospital and Health Service, Southport, QLD, Australia; ^2^ Faculty of Health Sciences and Medicine, Bond University, Robina, QLD, Australia; ^3^ Departments of Psychiatry and Pediatrics, Center for the Study and Prevention of Suicide, University of Rochester, New York, NY, United States; ^4^ Mental Health and Specialist Services, Metro North Hospital and Health Service, Herston, QLD, Australia

**Keywords:** zero suicide framework, suicide, prevention, pathway, youth, child, mental health

## Abstract

Suicide in children is a significant and growing problem. The “zero suicide” framework (ZSF) is one approach to suicide prevention used in health services for adults and children. This paper reports on the introduction of the first suicide prevention pathway (SPP) based on ZSF at a Child and Youth Mental Health Service (CYMHS) in Australia. It begins by describing the adaptations made to elements of the SPP originally designed for adults to meet the needs of children. Lessons learned in applying the SPP in the service are then discussed. The aim is to inform and improve practice in the use of zero suicide approaches in child and youth mental health settings in Australia and worldwide.

## 1 Introduction

### 1.1 Suicide in children

Suicide attempts and suicidal ideation among children (people aged ≤17 years) are significant and growing problems. In large American and European school samples, up to 39.4% of children reported experiencing suicidal thoughts, and up to 9.0% had made at least one suicide attempt (1, 2). Preventing and addressing suicidal ideation and attempts in children is, therefore, a priority. Systematic reviews consistently show that school-based interventions (e.g., suicide education, counselling) are moderately effective in reducing suicidal ideation and suicide attempts (3, 4). Other community-based interventions (e.g., support for young people bereaved by a suicide) and many therapeutic clinical interventions do not have consistent and/or sustained effects (3–5).

### 1.2 The zero suicide framework and suicide prevention pathway in Australia

One strategy for suicide prevention in health care settings, for adults and children, is the zero suicide framework (ZSF), developed by the National Action Alliance for Suicide Prevention (6). The framework involves suicide-specific practices which are delivered through whole systems of care and aim to continuously improve service access, quality, and safety (7, 8). It is a systems approach to suicide prevention that focuses on understanding the suicide event, formulating individualised risk, delivering first line interventions and follow-up, and drawing on the child’s strengths and resources. It also endorses support for staff through training and ongoing learning within services, and via evaluation of care and incidents within a Restorative Just Culture framework (9). The approach is rooted in a service culture that does not accept suicide as an outcome.

The ZSF and its clinical approach, the suicide prevention pathway (SPP), are new to the Australian setting and were first implemented at Gold Coast Mental Health and Specialist Services (GCMHSS) in Queensland, Australia in 2016 alongside the introduction of Australia-wide suicide prevention strategies (10). Implementation occurred in response to a review of increases in suicides in the service, and broader challenges around the delivery of suicide prevention interventions (11).

The GCMHSS sees >5,400 suicidal presentations each year via its two hospital emergency departments (EDs) (12). Young people aged 15-24 years account for 37.4% of suicidal presentations by females and 28.1% of presentations by males to GCMHSS (12). Children aged ≤17 years of age who engage in a suicide attempt or are deemed at risk of suicide by family or professionals are directed to the Child and Youth Mental Health Service (CYMHS) within GCMHSS. This service provides 24-hour multidisciplinary care to children who are experiencing severe and/or complex psychological, emotional, or behavioural problems, including (but not limited to) suicidality (13, 14).

The aim of this paper is to describe the adaptations made to the elements of the SPP originally designed for adults to meet the needs of children aged ≤17 years of age. Key lessons learned in applying the SPP in a children’s mental health setting are then presented. The intention is to inform and improve the use of zero suicide approaches in child and youth mental health settings globally.

## 2 The GCMHSS suicide prevention pathway for children

**Figure 1:**
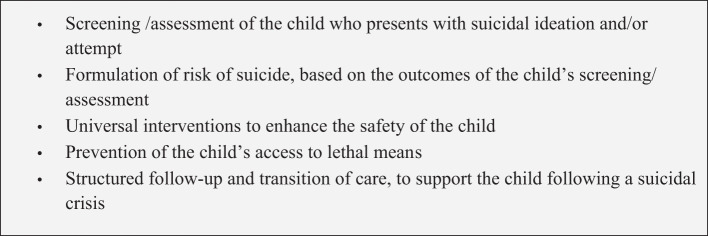
Key components of the SPP for children at the GCMHSS.

In this section we describe how the components of the SPP originally designed for adults have been adapted to meet the needs of children. We describe our experience and observations in using the SPP’s tools and highlight specific aspects to consider when providing care to suicidal children and their caregivers. **Figure 1** lists the key components of the SPP for children at the GCMHSS:

The components of the SPP are similar for children and for adults. However, the way the SPP is implemented differs between children and adults. For example: a child’s participation in components such as safety planning increases as their age and capacity does. Where a child does not have the capacity to participate or is too distressed to engage fully in components such as safety planning, parents/carers are more involved. We also acknowledge here that many components of the SPP, and the underlying philosophy, are fundamentally similar to other approaches to youth suicide prevention, such as SAFETY-Acute (15).

### 2.1 Screening

Screening to identify children at risk of suicide is an essential first component of the Zero Suicide framework. However, a standardised screening tool is not used to determine access to interventions or to predict an individual’s risk. At the GCMHSS CYMHS, screening and assessment for suicidality is undertaken using the Chronological Assessment of Suicidal Events (CASE) approach.

The CASE approach assists a clinician to explore suicidal ideation, planning, behaviours, and intent with a child, while maximising engagement with them (16). A strong therapeutic alliance and collaborative, non-judgmental stance is key. The CASE approach complements the broader comprehensive psychiatric assessment that considers risk/protective factors and warning signs (16).

With children, the CASE approach time interval format is applied more fluidly to enhance engagement. The child is supported to take control and to guide the process of disclosure, while the clinician gently navigates the conversation to ensure all timeframes are covered. Some of the questioning techniques in the CASE approach can be implemented with children with good effect. For example, the subtle shift of a closed questioning style (e.g., *“Have you thought of ways to end your life?”*) to a gentle assumption (e.g., *“What ways have you thought of to end your life?”*) may help a child to feel more comfortable discussing their methods. Open-ended questioning encourages clinically rich data, which is vital to informing safety planning. It also assumes that the young person is thinking of means of suicide, which gives them permission to share this with the clinician. The use of the behavioural incident techniques (e.g., fact finding, sequencing) is often well-received by children, who feel as though the clinician is comfortable with discussing their suicidality.

Consideration should be given to the power differences between the clinician and the child, along with the potential suggestibility of some children (e.g., the technique of denial of the specific and symptom amplification). Specific consideration should be given to younger children and those with developmental difficulties due to their potential suggestibility. The relevant CASE question has been altered to either include visual scales or by adding context (e.g., by asking the young person, *“On your worst day, do you think about suicide when you wake up? When you are at school? When you are with your friends? When you are at home with mum and dad?”*). A more narrative approach and interview style is helpful for younger children and for children with Aboriginal and/or Torres Strait Islander heritage, ensuring the timeframes are still woven into the assessment.

When working with children, the involvement of parents/carers in the CASE assessment is essential to ensuring comprehensive information-gathering. Parent/carer involvement is a strong protective factor for children who have attempted suicide (17). Parents/carers can provide valuable information about the observed amount of thinking, planning, or actions taken in relation to suicidal ideation that may reflect the intensity of the actual suicidal intent. Our clinicians identified that suicide attempts in children are frequently impulsive and reactive to feeling overwhelmed by shame, loss of control, hopelessness, and sadness, and interpersonal difficulties, especially with family/peers. We find that the time spent by a child on suicidal planning is, often, a more reliable reflection of the seriousness of their intent and of the proximity of their desire to proceed on that intent, than is the stated intent.

Where possible, and providing the clinician has consent, interviews should be conducted with the child without their parent/carer present. Our clinicians have observed that children often feel more comfortable to disclose risk without their parent/carer present. Interviews need to be conducted with the parent/carer separately to obtain information that may not be appropriate to discuss with the child. Subsequently, collaborative discussion with the child and their parent/carer are essential. This encourages understanding of the child’s suicidal thoughts and behaviours, equips the parent/carer with the necessary insight to provide adequate support, and is essential in informing safety planning. Equipping the parent/carer might involve, for example, providing psychoeducation on suicide, engaging them in safely planning, recognizing when their child is distressed and at risk of harming themselves, and supporting them to proactively restrict lethal means.

In our experience, in comparison to working with adults, using the CASE approach with a child is a more time-intensive process. As a service we learned to take younger children’s (<12 years) behavioural and verbal statements of hopelessness and wishing to die seriously when utilising this approach, whereas before these reactions were all too often deemed to be the child “acting out”.

**Figure 2:**
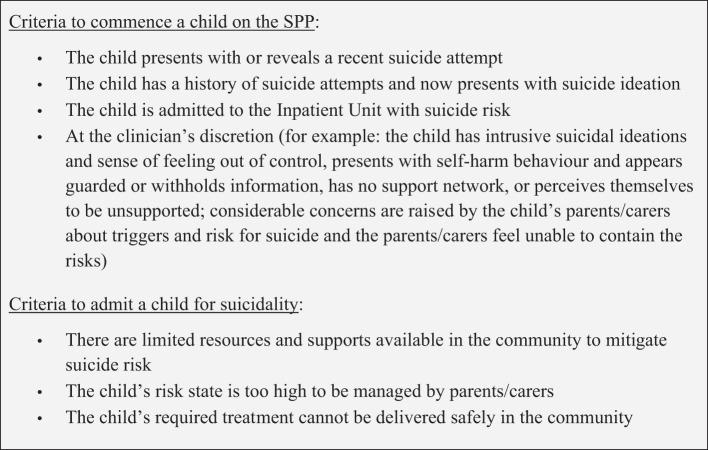
Criteria used at CYMHS to commence a child on the SPP and for admitting a child as an inpatient for suicidality.

**Figure 2** lists the criteria for commencing a child on the SPP at CYMHS:

### 2.2 Risk formulation

Categorical suicide risk stratification based on yes/no questions is widely accepted as inadequate in predicting future risk of suicide, and unreliable in determining who should and should not receive care (18–21). However, alternative approaches are limited and lack an evidence base. It has been argued that engagement, building a strong therapeutic relationship, understanding the context such as precipitating and perpetuating factors of a suicide attempt, and developing a management plan in collaboration with the consumer – in this case, the child – to mitigate and manage risks is of more value and importance than risk categorisation (18, 20, 22).

When implementing the SPP with children, we extended on this idea, recognising the importance of parents/carers as the key support system. Parents/carers are vital in understanding the child’s suicide attempt, developing a management plan collaboratively with the child, and engaging in a therapeutic alliance. Equally, including a child’s wider identified support network in safety planning is helpful.

The prevention oriented risk formulation (23) synthesises information gathered through an assessment of the child and their support network. Risk status and risk state are two ways of contextualising risk. ‘Risk status’ describes a judgment of the child’s risk relative to others in a specific population (e.g., the local general population of the same age and/or same developmental age) or setting (e.g. population attending local community or inpatient mental health settings). ‘Risk state’ describes a judgment about risk in relation to the child’s own baseline or other selected time points. Clinicians arrive at judgments about risk status and state through reports from the child, reports from their parent/carer, and information from clinical observations.

More important than the determination of risk status/state is communicating the factors and thinking behind the determination. Prevention-oriented risk formulation then directs attention to identifying available internal resources (individual) and external resources (parents’/carers’ and others’), and foreseeable changes which might lead to a rapid increase or decrease in risk (23). One of the foreseeable changes should always be the driver/s of the suicide attempt, as addressing driver/s is an important part in planning for effective treatment (24). Importantly, the risk formulation is not used for predictive purposes or to determine acceptability for treatment, but rather for communication within the team, with the parent/carer, and with the child. It enables broader understanding of the issues for the child so that an individual, forward-looking, and collaborative plan can be developed.

### 2.3 Universal interventions

After assessment, safety planning with the child and their parent/carer, and risk formulation, a child will be offered care through the SPP. This will include universal interventions, which aim to enhance safety as part of an individualised care plan. ED settings are focus areas for brief interventions (25). Universal interventions identified as deliverable in busy ED settings include safety planning intervention (SPI), counselling on restricting access to lethal means (embedded in SPI), crisis numbers (delivered as part of SPI), brief patient and carer information, and arranging rapid follow up (26).

Children who present with acute suicidality and a risk profile above baseline can be challenging to engage in assessment, treatment, and follow up care. In our experience, when in crisis, children frequently struggle to talk about their feelings and thoughts. Parent/carers can also be significantly overwhelmed, distressed, anxious, and unable to meet the needs of their child. To manage the stress, both the child and the parent/carer might minimise the suicidal event, or they might be unable to reflect and develop insight into what triggered and maintained or what might resolve it. Hence, support through a structured and brief process to problem-solve safety issues is beneficial. Issues identified through this process are revisited and further explored during the follow-up appointments.

Suicidal thoughts often fluctuate over time (27). Children who are given skills and strategies for future use may be able to resist or delay acting on suicidal thoughts until they subside or care can be accessed. The SPI is a prioritised set of coping skills and supports, developed in collaboration with the child and a parent/carer or other support person, used for this purpose (28).

The SPI includes processes to identify warning signs, internal coping strategies, social contacts to distract from suicidal thoughts, social and professional supports, and strategies to restrict access to lethal means of suicide. There is growing evidence that the SPI improves consumer engagement, helps in resolving suicidal crises (25, 29), and reduces repeated self-harm and suicide risk (29, 30).

The SPI was modified for the SPP to include two additional questions about the drivers of suicide and solutions to these, contact details of the consumer and an alternative contact, and information on local and national 24-hours crisis numbers. A child-friendly version was developed to help children in crisis to overcome barriers to reflect, communicate, and problem-solve. The redesign involved input from children, CYMHS multidisciplinary clinicians, and other stakeholders. It includes pictures, colours, prompts to stimulate reflection and problem-solving and more child-friendly language throughout (e.g., a shift from *‘Internal coping strategies’* to *‘Things I can do to help me get through this’*). It also includes child-specific crisis numbers, webchat options, and apps to support safety planning. When delivering the SPI, clinicians employ features of motivational interviewing, which has been used with success in youth suicide prevention (31, 32), to encourage engagement.

Clinicians also developed an approach to engaging parents/carers as part of the SPI. This is collaborative, with emphasis on the child feeling heard, understood, and supported in a way that they perceive to be helpful. It provides an opportunity to bring the child and their support system closer, and to safety plan with the support system in times when the child themselves might struggle.

### 2.4 Preventing access to lethal means

Lethal means counselling is an essential component of safety planning, and it has been shown to reduce the risk of a suicide attempt and death (33). It is conducted with both the child and the parent/carer and based on the information obtained through earlier interviews. It is imperative that counselling is followed up with a phone call to the parent/carer to ensure the agreed actions were, or are being, implemented. A child in crisis often places the whole family system in crisis, and this can impact a family’s ability to retain information and take the appropriate steps to meet the needs of the child. Hence, it is paramount to extend ongoing support to the family and broader networks.

One of the complexities of safety planning occurs when access to a method of high lethality has been identified by the clinician but is not recognised by the child (for example, when a child lives on a rural property and has access to firearms but the child has not identified this as a method of suicide). Often children are not as advanced as adults with identifying methods, yet these must be included in safety planning. In such instances, separate conversations need to be held with the parent/carer to ensure safety planning has occurred. A parent/carer version of a safety plan might be drafted.

### 2.5 Structured follow-up and transition of care

Effective transitions of care are of central importance in the ZSF, considering the elevated risk of suicide in the post-discharge period (34), however engaging with consumers following a suicide attempt can be very challenging. In children specifically, only 76% may attend follow-up outpatient treatment appointments (35). In adults, strategies such as scheduling the first appointment within two to three days, and intensive outreach in the post discharge period, may improve follow-up (36).

Community follow-up requires a consistent, structured approach. It include clinical activities such as Mental State Examination with a focus on mood and reassessment of suicidality, the child’s risk state and status and foreseeable changes, available resources, collaborative review and revision of the safety plan, collaborative development of a care plan, identification of barriers to implementing the care plan, and agreement on further follow-up care. A care plan should be completed collaboratively with the parent/carer and should involve the driver for the suicide attempt and the strategies to mitigate risk. The clinician explores whether ongoing care is necessary and which services are best suited, and provides a seamless handover where the child has their first appointment with the next provider prior to closure of care under the SPP. Closure of the SPP is not a process-driven decision (e.g. as the child has their first appointment with the next provider); sometimes, there is no onward referral.

## 3 Discussion

### 3.1 Learnings from SPP implementation

**Figure 3:**
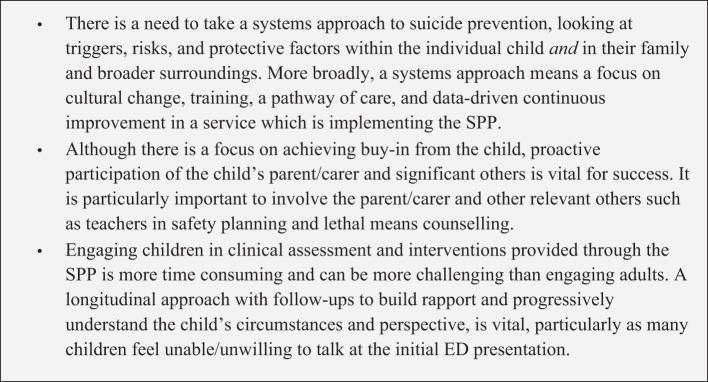
Learning highlights from implementing the SPP at the GCMHSS CYMHS.

Sustained change in large health systems is challenging to bring about, and change initiatives have high failure rates (37). We have outlined the steps taken to implement and embed a significant change in clinical approach to suicide prevention in a large child and youth mental health service in Australia. Implementing and maintaining fidelity to such a change in the absence of a significant increase in clinical resources can be achieved but has not been without challenges. **Figure 3** shows the learning highlights from implementing the SPP at the GCMHSS CYMHS:

### 3.2 Conclusions

Globally, suicide in children is a significant and growing problem. The Zero Suicide Framework (ZSF) is one approach to suicide prevention adopted in health services for adults and children. This paper reports on the introduction of the first Suicide Prevention Pathway (SPP) based on ZSF at a Child and Youth Mental Health Service in Australia. It describes the adaptations made to elements of the SPP originally designed for adults to meet the needs of children, and presents the lessons learned. It shows that a standardised approach to suicide prevention improves consistency in the delivery of first line interventions and, hence, has the potential for significant positive clinical outcomes.

## Data availability statement

The raw data supporting the conclusions of this article will be made available by the authors, without undue reservation.

## Ethics statement

The studies involving humans were approved by Gold Coast Health Human Research Ethics Committee (HREC) Reference LNR/2018/QGC/47473, 11 October 2018. The studies were conducted in accordance with the local legislation and institutional requirements. The ethics committee/institutional review board waived the requirement of written informed consent for participation from the participants or the participants’ legal guardians/next of kin because This study is exempt from ethical review as it involves routine data collection undertaken for clinical quality assurance.

## Author contributions

GB: Formal analysis, Investigation, Methodology, Project administration, Visualization, Writing – original draft, Writing – review & editing. LM: Formal analysis, Investigation, Methodology, Project administration, Visualization, Writing – original draft, Writing – review & editing, Validation. DJ: Data curation, Writing – original draft, Writing – review & editing. SM: Data curation, Methodology, Writing – original draft, Writing – review & editing. PW: Resources, Software, Writing – original draft, Writing – review & editing. SC: Resources, Software, Writing – original draft, Writing – review & editing. HJ: Resources, Software, Writing – original draft, Writing – review & editing. AP: Conceptualization, Validation, Writing – original draft, Writing – review & editing. CS: Conceptualization, Methodology, Validation, Writing – original draft, Writing – review & editing. MW: Conceptualization, Writing – original draft, Writing – review & editing. KT: Conceptualization, Methodology, Writing – original draft, Writing – review & editing. SW-M: Conceptualization, Investigation, Methodology, Supervision, Validation, Writing – original draft, Writing – review & editing.
